# Florid cystic endosalpingiosis: Diagnostic challenges and management in the spectrum of müllerian anomalies – A case report

**DOI:** 10.1016/j.ijscr.2025.110840

**Published:** 2025-01-08

**Authors:** Ghaddab Imen, Bergaoui Haifa, Toumi Dhekra, Cheikh Mohamed Chayma, Bayar Amal, Faleh Raja

**Affiliations:** a Department of Gynecology and Obstetrics, Fatouma Bourguiba University Hospital, Tunisia; b Department of Gynecology and Obstetrics, Farhat Hached University Hospital, Tunisia

**Keywords:** Adnexa, Endosalpingiosis, Menopausal woman, Benign disorder, Case report

## Abstract

**Introduction and importance:**

Cystic endosalpingiosis is a rare, benign condition characterized by the presence of fallopian tube-like epithelium outside the fallopian tubes. It predominantly affects menopausal women and is often asymptomatic. Florid cystic endosalpingiosis, an unusual form, can mimic malignant ovarian masses, making accurate diagnosis crucial.

**Case presentation:**

We present a 48-year-old postmenopausal woman with chronic pelvic pain, found to have a suspicious multicystic mass during ultrasound. Laparoscopic exploration revealed extensive cystic endosalpingiosis involving the uterine surface and left adnexa. Histopathological analysis confirmed the diagnosis.

**Clinical discussion:**

Endosalpingiosis often presents a diagnostic challenge due to its nonspecific symptoms and potential to mimic neoplastic conditions. This case underscores the need for histological examination to differentiate between benign and malignant pelvic masses. Surgical resection is recommended for symptomatic cases, while asymptomatic ones may not require intervention.

**Conclusion:**

Florid cystic endosalpingiosis should be considered in the differential diagnosis of multicystic pelvic masses in menopausal women. Accurate histological diagnosis is essential to exclude malignancy and guide appropriate management.

## Highlights

• Florid cystic endosalpingiosis mimics an ovarian cyst in menopausal women.
• It is often asymptomatic and found incidentally during surgery.
• Diagnosis relies on histopathology, highlighting tubal-like epithelium.
• Differentiation from malignancy requires biopsy due to tumor-like appearance.

## 1 Introduction

Cystic endosalpingiosis is a benign gynecological disorder in which cystic Müllerian tissue, resembling the structure of the fallopian tubes, is found outside of them, specifically in the pelvic and abdominal cavities [1].

It is an extremely rare condition that appears to afflict menopausal women in 40 % of instances and is associated with endometriosis lesions in 34.5 % [1].

The symptoms of endosalpingiosis are not well established. Studies vary as to whether it causes pelvic pain, or if it is a pathology discovered incidentally during the exploration of pelvic pain, menstrual irregularities, or infertility [1].

Given the clinical heterogeneity of endosalpingiosis, this article presents a case, consistent with the SCARE guidelines 2023 [2] and demonstrates the importance of including a complete history and gynecologic examination in the clinical evaluation of chronic pelvic pain in postmenopausal women.

## 2 Case report

A 48-year-old female presented with a history of chronic pelvic pain progressively worsening over the last 12 months. The woman reported no signs of irregular vaginal bleeding, dyspareunia, or gastrointestinal or urinary problems. She had been menopausal for four years and had not undergone any hormonal therapy for her menopausal state. Her substantial medical history was limited to primary infertility. However, her surgical history was remarkable and included a laparoscopy performed 14 years earlier for a benign ovarian mass, during which no multiple cystic formations were discovered. She also had a hysteroscopic excision of an endometrial polyp nine years ago and a cholecystectomy at the age of 35.

Pelvic examination revealed a hard, non-tender, fixed mass measuring 7 cm in its largest diameter and lateralized to the left. A speculum examination revealed no cervical abnormalities but there was a firm, protruding 6 cm mass in the cul-de-sac of Douglas.

**Fig. 1:**
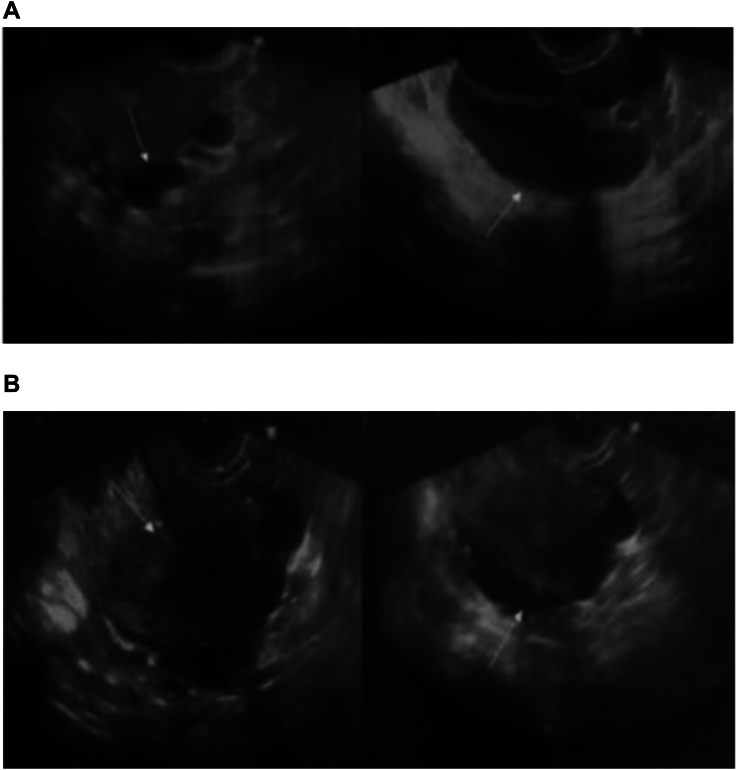
Transvaginal ultrasonography revealed a well-defined, round, multicystic mass measuring 6 cm, characterized by homogeneous cystic contents situated in the cul-de-sac. B: Multiple anechoic cystic formations were observed surrounding the fundus of the uterus.

Transvaginal ultrasound imaging displayed a well-circumscribed round multicystic mass (6 cm) with homogeneous cystic content located in the cul-de-sac. Multiple anechoic cystic formations around the fundus of the uterus (Fig. 1). Serum CA125 levels were normal.

Given her symptoms and ultrasound findings, she was admitted to our department for diagnostic laparoscopy of these suspicious masses after one week.

**Fig. 2:**
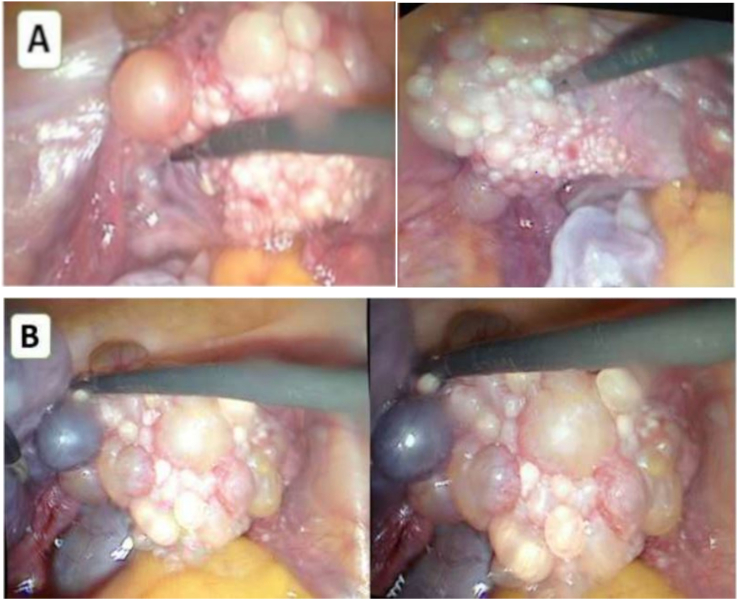
A: Laparoscopic visualization of cystic endosalpingiosis: lesions multiple cystic lesions on the surfaces of the left ovary, fallopian tubes, and uterus. B: A large 6 cm multilocular cyst with a bluish hue and a pedicle from the posterior wall of the uterus, mimicking a left ovarian cyst.

During laparoscopy, the fundus and part of the uterine body were extensively seeded with multiple translucent cystic structures resembling Morgagni's hydatids but with a firmer consistency. These cystic lesions were also located around the left adnexal area, encasing the ovary and fallopian tube into a 6 cm bluish cystic mass without exocytic vegetations (Fig. 2).

No cystic lesions were noted on the pelvic peritoneum, vesical peritoneum, or Douglas' pouch. The omentum, intestinal surface, and upper abdominal cavity were not involved. There were also no signs of pelvic endometriosis or adhesions. Samples of cystic fluid and some whole cysts were sent for histopathological examination.

Intraoperative studies, such as frozen section analysis or rapid cytological evaluation, were not performed. This decision was influenced by the limited availability of such diagnostic facilities in our institution, which primarily serves a resource-constrained setting. Despite these limitations, every effort was made to collect adequate samples for thorough postoperative histopathological analysis to ensure diagnostic accuracy.

We performed cyst resection of the cystic lesions, rather than total laparoscopic hysterectomy with bilateral salpingo-oophorectomy (TLHBSO).

Histological results revealed that the cyst walls were lined by both squamous and cylindrical ciliated epithelium resembling tubal type. The walls were surrounded by fibrous tissue containing fibroblastic cells and often by normal-looking myometrial tissue. Although some decidualized cells were present, no endometrial stroma was found. Cytology showed histiocytes and rare inflammatory cellularity.

The final diagnosis was florid cystic endosalpingiosis.

## 3 Discussion

Endosalpingiosis is an uncommon condition with an incidence of 7.6 % to 12.5 % [3]. This benign condition is distinguished by the development of cysts lined with ciliated cylindrical epithelium similar to that found in tubal type [3]. Sampson originally characterized endosalpingiosis in 1930, when he discovered a fallopian tube-like epithelium in the pelvis of women who had undergone salpingectomies or tubal ligations [4]. Endosalpingiosis cysts are most typically found in pelvic organs such as the uterine serosa, fallopian tubes, ovaries, round ligament, and bladder, but they have also been described on several other abdominal peritoneal and retroperitoneal surfaces, including retroperitoneal lymph nodes [5].

Pathogenically, some experts consider endosalpingiosis to be part of a group of embryological origin anomalies called “Müllerianosis” [6]. This condition involves the heterotopic presence of Müllerian-derived tissues in pelvic organs or distant locations. This may explain the concurrent presence of endosalpingiosis with endometriosis and endocervicosis [7].

During organogenesis, several genes from the WNT family, such as WNT4, are activated, producing the necessary signals for the development of Müllerian structures. Recent study has suggested that Müllerianosis may be caused by aberrant reactivation of these genes [8], resulting in the metaplasia of normal tissues such as the peritoneum. This would explain why these defects are spread across the pelvic and abdominal organs [5].

Furthermore, as with endometriosis, some studies link the lesions of endosalpingiosis to ectopic transport. As a result, any previous surgical intervention on the fallopian tubes or ovaries, regardless of the primary cause for the procedure, may lead to endosalpingiosis [7]. This was the case for our patient, who had previously had ovarian cystectomy via laparoscopic. Endosalpingiosis is frequently related, in 35% to 88% of cases, with chronic pelvic inflammation or distorted fallopian tube architecture (resulting from previous pelvic inflammatory disease or prior surgery) [5].

The clinical features of endosalpingiosis remain uncertain [4]. Unlike endometriosis, there is no significant link between infertility, chronic pelvic pain, and endosalpingiosis.

Endometriosis is symptomatic in most cases, while endosalpingiosis is typically an incidental finding. Both conditions can cause a variable spectrum of symptoms depending on the involved sites. The severity of symptoms is not directly related to the extent of the disease [8]. Common symptoms include: acquired dysmenorrhea, lower abdominal, pelvic, and back pain, dyspareunia, irregular bleeding, and infertility. Clearly, none of these symptoms are specific [8]. Therefore, a key challenge is to clinically differentiate it from endometriosis.

Transabdominal and transvaginal ultrasound are both effective in detecting florid cystic endosalpingiosis. It frequently appears as numerous cysts of varying dimensions with thin walls and uniform contents [9]. However, it is difficult to distinguish from other nonneoplastic peritoneal inclusion cysts, which should also be included in the differential diagnosis [10].

Endosalpingiosis is frequently diagnosed through surgical biopsy. Histologically, ES is characterized by the presence of cysts bordered by tubal-type epithelium free of cellular atypia and surrounded by non-endometrial fibrous stroma [1]. To date, the relationship between ES and gynecological malignancy is well established. Large series of ES have demonstrated that premenopausal women with endosalpingiosis are more likely to have a gynecological malignancy. Similarly, endosalpingiosis is often found concurrently with other serous pelvic neoplasias, such as borderline serous tumors, low- and high-grade serous adenocarcinomas [3].

This justifies a thorough intraoperative inspection of pelvic organs when endosalpingiosis is identified, and given the above-mentioned association and the difficulty in distinguishing florid cystic endosalpingiosis from other pelvic cystic neoplasias based on clinical features and overall examination, removal for histological evaluation is required to exclude malignant pathologies. On the other hand, particularly if histology confirms the benign nature of the lesions, there is no solid evidence to indicate a hysterectomy [7]. Treatment for endosalpingiosis is not always necessary, especially if the patient is asymptomatic. However, treatment is warranted in cases of endosalpingiosis causing chronic pelvic pain, dyspareunia, in the context of infertility, and in cases of suspicious ovarian cysts [12].

Postoperatively, patients commonly report mild pelvic discomfort and bloating, which generally resolve within a few weeks, mirroring findings in similar case reports. In some cases, transient symptoms such as mild abdominal pain or distension were noted, especially in patients who underwent surgical resection of cysts or adhesions. These symptoms are typically related to the healing process and the removal of cystic structures. While the recovery timeline may vary, the overall symptomatology in our case was consistent with what has been described in other case reports of endosalpingiosis, where symptoms are often transient and non-specific [13,14].

Similar to endometriosis, endosalpingiosis can be treated by surgical resection, although this is not a cure but the removal of tissues, cysts, and adhesions can help significantly reduce symptoms such as pelvic pain [10]. To date, there is no proven association between endosalpingiosis and infertility, and patients diagnosed without concurrent endometriosis are generally treated as unexplained infertility [12]. Some surgeons believe that progesterone therapy may also help alleviate symptoms [15]. In cases that are particularly sensitive to hormonal influences, dietary estrogen can cause significant disruptions. Similarly to endometriosis management, it is recommended that women adhere to a diet low in estrogen [15].

## 4 Conclusion

To summarize, cystic endosalpingiosis is a benign condition that should always be investigated when diagnosing a multicystic pelvic tumor. It poses a diagnostic challenge due to its subtle clinical presentations, which may include mild pelvic discomfort, bloating, and occasionally, abdominal pain or distension, especially following surgical procedures. Patients may also present with non-specific symptoms such as irregular menstrual cycles or infertility. Accurate histology confirmation is essential for a definitive diagnosis. Given its potential association with gynecological malignancies, medical professionals must be vigilant and conduct thorough evaluations when encountering pelvic cystic formations. Accurate diagnosis and management can avoid unnecessary surgical procedures and improve patient outcomes. This case underscores the importance of recognizing endosalpingiosis as a distinct entity in the spectrum of Müllerian anomalies, highlighting the necessity for awareness and specialized knowledge in its management.

## Author contribution

**Cheikh Mohamed Chayma**: Conceptualization, Methodology, Software.

**BayarAmal**: Data curation, Writing- Original draft preparation.

**Bergaoui Haifa**: Visualization, Investigation.

**Toumi Dhekra**: Software, Validation.

**Ghaddab Imen**: Writing- Reviewing and Editing.

**Faleh Raja**: Supervision.

## Patient consent

Written informed consent was obtained from the patient to publish this case report and accompanying images. On request, a copy of the written consent is available for review by the Editor-in-Chief of this journal.

## Ethical approval

As a case report, it is exempted from ethical approval by the Institutional Board of Review, Monastir University Hospital, Monastir.

## Guarantor

Ghaddab Imen.

## Research registration number

Not applicable.

## Funding

This research did not receive specific grants from the public, commercial or not-for-profit sectors.

## Conflict of interest statement

The authors do not have any conflicting interests to declare.

## References

[1] Junsik P, M.D., Tae-Hee Kim, Ph.D., Hae-Hyeog L, M.D., Ph.D., Soo-Ho C et al. Endosalpingiosis in postmenopausal elderly women. J. Menopausal Medicine 2014;20:32–34. 10.6118/jmm.2014.20.1.32 PMC4217568 25371889

[2] Sohrabi C., Mathew G., Maria N., Kerwan A., Franchi T., Agha R.A. (2023). The SCARE 2023 guideline: updating consensus Surgical CAse REport (SCARE) guidelines. Int. J. Surg. Lond. Engl..

[3] Katharine E., Kathryn T., Anicka S., Kevin E., Michelle D., William W., Michael M. (2016). Endosalpingiosis: more than just an incidental finding at the time of gynecologic surgery?. Gynecol. Oncol..

[4] Prentice L., Stewart A., Mohiuddin S., Johnson N.P. (2012). What is endosalpingiosis?. Fertil. Steril..

[5] José M.R., Loida P.B., Beatriz M.B., Domingo D.R. (2016). Florid cystic endosalpingiosis (Müllerianosis) in pregnancy. Case Rep. Obstet. Gynecol..

[6] Batt R.E., Yeh J. (2013). Müllerianosis: four developmental (embryonic) Müllerian diseases. Reprod. Sci..

[7] Meilin Y., Yuqin L., Mengkun Ch., Jowei Ch., Fu-Tsai K. (2019). Uterine endosalpingiosis: case report and review of the literature. Taiwan. J. Obstet. Gynecol..

[8] Scheel A.H., Frasunek J., Meyer W., Ströbel P. (2013). Cystic endosalpingiosis presenting as chronic back pain, a case report. Diagn. Pathol..

[9] Felix W.S., Eric T.C.L. (2016). Florid cystic endosalpingiosis presenting as an ovarian cyst in a postmenopausal woman. Gynecol. Minim. Invasive Ther..

[10] Baker P.M., Clement P.B., Young R.H. (2014). Selected topics in peritoneal pathology. Int. J. Gynecol. Pathol..

[12] Nixon K.E., Kenneth S.J., Bakkum-Gamez J.N. (2018). Florid cystic endosalpingiosis with uterine preservation and successful assisted reproductive therapy. Gynecol. Oncol. Rep..

[13] Abdi F., Sharifi N. (2020). Postoperative outcomes following laparoscopic surgery for endosalpingiosis: a case report. Int. J. Surg. Case Rep..

[14] Patel S., Gupta R. (2021). Postoperative symptoms after laparoscopic management of endosalpingiosis: a case series. Int. J. Surg. Case Rep..

[15] Endosalpingiosis. Wikipedia, the free encyclopedia.

